# The calcium-activated potassium channel KCa3.1 is an important modulator of hepatic injury

**DOI:** 10.1038/srep28770

**Published:** 2016-06-29

**Authors:** Linda Sevelsted Møller, Annette Dam Fialla, Robert Schierwagen, Matteo Biagini, Christian Liedtke, Wim Laleman, Sabine Klein, Winfried Reul, Lars Koch Hansen, Maj Rabjerg, Vikrant Singh, Joaquin Surra, Jesus Osada, Roland Reinehr, Ove B. Schaffalitzky de Muckadell, Ralf Köhler, Jonel Trebicka

**Affiliations:** 1 Department of Medical Gastroenterology and Hepatology, Odense University Hospital, Odense, Denmark; 2 Institute of Molecular Medicine, University of Southern Denmark, Odense, Denmark; 3 Department of Internal Medicine I, University of Bonn, Bonn, Germany; 4 Department of Pathology, Odense University Hospital, Odense, Denmark; 5 Department of Internal Medicine III, University Hospital RWTH Aachen, Aachen, Germany; 6 Department of Liver and Biliopancreatic disorders, University of Leuven, Leuven, Belgium; 7 Department of Medical Gastroenterology and Hepatology, Vejle Hospital, Vejle, Denmark; 8 Department of Pharmacology, University of California, Davis, California, USA; 9 Departament de Producción Animal, Escuela Politécnica Superior, Huesca, Spain; 10 Departamento Bioquímica y Biología Molecular y Celular, Facultad de Veterinaria, Instituto de Investigación Sanitaria de Aragón (IIS), Universidad de Zaragoza-CIBEROBN, Zaragoza, Spain; 11 Elbe-Elster Klinikum, Krankenhaus Herzberg, Herzberg, Germany; 12 Aragon Institute of Health Science I CS, Zaragoza, Spain

## Abstract

The calcium-activated potassium channel KCa3.1 controls different cellular processes such as proliferation and volume homeostasis. We investigated the role of KCa3.1 in experimental and human liver fibrosis. KCa3.1 gene expression was investigated in healthy and injured human and rodent liver. Effect of genetic depletion and pharmacological inhibition of KCa3.1 was evaluated in mice during carbon tetrachloride induced hepatic fibrogenesis. Transcription, protein expression and localisation of KCa3.1 was analysed by reverse transcription polymerase chain reaction, Western blot and immunohistochemistry. Hemodynamic effects of KCa3.1 inhibition were investigated in bile duct-ligated and carbon tetrachloride intoxicated rats. *In vitro* experiments were performed in rat hepatic stellate cells and hepatocytes. KCa3.1 expression was increased in rodent and human liver fibrosis and was predominantly observed in the hepatocytes. Inhibition of KCa3.1 aggravated liver fibrosis during carbon tetrachloride challenge but did not change hemodynamic parameters in portal hypertensive rats. *In vitro*, KCa3.1 inhibition leads to increased hepatocyte apoptosis and DNA damage, whereas proliferation of hepatic stellate cells was stimulated by KCa3.1 inhibition. Our data identifies KCa3.1 channels as important modulators in hepatocellular homeostasis. In contrast to previous studies *in vitro* and other tissues this channel appears to be anti-fibrotic and protective during liver injury.

Hepatic fibrosis is the common response to any form of chronic liver injury and upon progression it undermines liver function and initiates portal hypertension. Activated and myofibroblast-like hepatic stellate cells (HSC), instigated and perpetuated by inflammation secondary to hepatocellular damage, are considered the main cellular source for the extracellular matrix during liver fibrogenesis^1^. Nevertheless, quiescent HSC were reported to contribute to progenitor cells and liver regeneration^2^. Hepatocytes show a high regenerative potential upon injury and frequently react with adaption of the cell volume, which is suggested to be controlled by the calcium-activated potassium channel KCa3.1^3^. This channel is activated by Ca^2+^ binding to constitutively bound calmodulin, and the K^+^-efflux and concomitant water efflux leads to cell volume reduction. In hepatoma cell-lines KCa3.1 activation seems to be crucial for volume restoration after cell swelling caused by hypotonic stress and thereby for hepatocyte volume control^3^.

Besides its role in cell volume regulation, the KCa3.1 channel modulates other cellular processes such as cell proliferation and endothelium-dependent vasorelaxation^4^,^5^,^6^,^7^,^8^, and was shown to be pathomechanistically implicated in renal and pulmonary fibrosis^9^,^10^,^11^,^12^. Indeed, KCa3.1 deficiency in mice blunted renal fibrosis^13^, and the KCa3.1 inhibitor TRAM-34 was shown to suppress fibrogenic properties of myofibroblasts *in vitro*^14^. Yet, the pathomechanistic roles of KCa3.1 in liver fibrosis especially in experimental studies and in human situation remains unclear, especially in regards to the potential effect of the channel on the high hepatic regenerative potential compared to other parenchymal organs.

Phenotypically, KCa3.1 deficient mice are characterized by a sizable splenomegaly, which is linked to KCa3.1’s well-established effect on erythrocyte volume regulation^15^. Furthermore, the mice have a mild arterial hypertension^16^, are hyperactive and have higher bodyweights than wild type littermates^17^.

In this study, we hypothesized pathomechanistic roles of KCa3.1 in liver injury and fibrosis and tested whether genetic deficiency or pharmacological blockade alters fibrotic remodelling and expression of profibrotic mediators in experimental rodent models.

## Materials and Methods

### Human liver specimen

Liver biopsies were performed using the Menghini method (17G Menghini needle, Hepafix, Braun, Germany). Biopsies were collected from 54 patients at the Department of Medical Gastroenterology, Odense University Hospital. We included patients, who for clinical reasons were scheduled to undergo liver biopsy, and used the excess tissue for further analyses. Oral and written informed consent was given prior to biopsy, and all experiments were performed in accordance with the approved guidelines and regulations. The study was approved by The Regional Scientific Ethical Committees for Southern Denmark (case number S-20110115) and was in agreement with the 1975 Declaration of Helsinki.

Samples were immediately stored in 4% formalin and sent to routine histological staining and evaluation. METAVIR score was evaluated blinded by a hepatopathologist and performed on all biopsies. Patients were classified into groups based on fibrosis score; non-fibrotic (n = 12), F1–F2 fibrosis (n = 20) and F3–F4 fibrosis (n = 22) (Table 1).

### Animal studies

All animals within this study received human care according to the criteria outlined in the Guide for the Care and Use of Laboratory Animals. Animals were housed in a room with controlled temperature and humidity in a 12-hour light cycle. KCa3^−/−^ mice, also known as Kcnn4 deficient mice, were obtained from own breeding colonies^5^. The genetic background for the KCa3.1 deficient mice is C57BLJ/6 × 129 Ola. KCa3.1 knockout mice were bred by homozygote breeding and KCa3.1 deficiency was furthermore verified by quantitative polymerase chain reaction (qPCR). We used KCa3.1^+/+^ or KCa3.1^+/−^ animals as wt controls. Experimental protocols were approved by the Danish Animal Experience Inspectorate (2012-15.2934-00264).

For rat studies we used Sprague-Dawley rats. The responsible committee for animal studies in North Rhine-Westphalia approved this part of the study (LANUV 8.87-50.10.31.08.28).

#### Mouse models of fibrosis

We used KCa3.1^−/−^ mice and corresponding wt littermates at age 12–16 weeks. The mice were challenged with carbon tetrachloride (CCl_4_) (1 ml/kg *i.p.*) twice weekly. Animals were grouped according to a CCl_4_ exposure time of 8 weeks to induce fibrosis^18^ and 12 weeks to induce advanced fibrosis or cirrhosis^19^. For each exposure group we included 8–10 wt and 8–10 KCa3.1 knockout mice. Age and sex matched untreated wt and KCa3.1^−/−^ mice (n = 10 wt, 10 KCa3.1^−/−^) served as controls.

#### Rat models of fibrosis

For CCl_4_ intoxication in rats we exposed rats (n = 4) with an initial body weight of 100–120 g to CCl_4_ inhalation 1 l/min for 14–16 week as described previously^20^,^21^. For thioacetamide (TAA) induced injury we administered TTA in 200–250 g rats (n = 4) in their drinking water for 18 weeks as previously described^22^.

Bile duct ligation (BDL) and transection was performed under anaesthesia with ketamine/xylazine as previously described^20^,^21^. Hemodynamic experiments were conducted four weeks after BDL surgery in 3 rats.

### Treatment with the KCa3.1-blocker Senicapoc

For pharmacological studies, wt mice were used. Animals were exposed to CCl_4_ for 12 weeks, and allocated to receive either normal chow (placebo) (n = 10) or the oral KCa3.1 antagonist Senicapoc (n = 10). Senicapoc (ICA-17043) was synthesized as previously described^23^ and animals received a dosage of 30 mg/kg/day mixed in the chow. Senicapoc was administered in the fodder and initiated 48 hours prior to the first CCl_4_ injection. Food intake was measured weekly until termination. Prior to termination, animals were anaesthetised with fentanyl/fluanisone and midazolam, blood was collected through penetration of the retro-orbital sinus, and plasma was separated by centrifuge and stored at −80 degrees Celsius until analysed. The concentration of Senicapoc was measured by mass spectrometry (TSQ Quantum Access MAX, ThermoFisher Scientific, Waltham, MA, USA).

### Hemodynamic studies

*In vivo* measurements of portal pressure (PP), mean arterial pressure (MAP) and heart rate (HR) were performed in BDL (n = 3) and CCl_4_ exposed (n = 3) rats during anaesthesia with ketaminen/xylazine (78 and 12.5 mg/kg *i.m.* respectively) as previously described^20^,^21^,^24^,^25^. Briefly, following midline laparotomy, a PE-50 catheter was inserted into the ileocecal vein and progressed to the portal vein. This was followed by insertion of a similar catheter in the femoral artery for MAP measurement. Both catheters were connected to a pressure transducer (Hugo Sachs Electronic, March-Hugstetten, Germany). Hemodynamic stabilization was awaited before proceeding and subsequently 100 nM of the KCa3.1 antagonist TRAM-34 was injected as bolus. Continuous monitoring of HR, MAP and PP was performed for the following 20 minutes.

### Quantitative RT-PCR

For murine samples RNA was isolated using TRIZOL reagent (Invitrogen, United Kingdom) and DNAse digestion using RNase free DNase 1 (Thermo Scientific, CA, USA) according to manufactures instructions. cDNA was synthesized with iScript cDNA Synthesis Kit (Bio-Rad, CA, USA). qRT-PCR was performed using SYBR Green Supermix (Bio-Rad, CA, USA) on the Stratagene MX3000 qPCR instrument (Agilent Technologies, CA, USA). Glyceraldehyde-3-phosphate dehydrogenase (GAPDH) served as endogenous control.

For gene expression analyses in rat and cell studies, RNA isolation, reverse transcription and detection by RT-PCR were performed by TaqMan method as described previously^20^,^21^,^26^. 18S rRNA served as endogenous control.

Results are expressed as 2^−ΔΔCt^, and express the x-fold increase of gene expression compared to the control group, and in murine studies results were compared to untreated wt.

### Western blotting

Western blot was performed on samples from untreated and fibrotic wt and KCa3.1^−/−^ mice (12 weeks CCl_4_ exposure) for Proliferating Cell Nuclear Antigen (PCNA; Santa Cruz-Biotechnology, Santa Cruz, USA). Proto-oncogene tyrosine-protein kinase Src (c-Src) activity was analysed by phosphorylation either at Thr418 or at Thr530 (both Santa Cruz-Biotechnology, Santa Cruz, USA). Activation of c-Jun N-terminal kinases 1 and 2 (pJNK1&2; Life technologies, Darmstadt, Germany) and total JNK (Santa Cruz-Biotechnology, Santa Cruz, USA) was assessed by their phosphorylation at Thr183, respectively Tyr185. GAPDH served as endogenous control.

Snap frozen liver samples were processed as previously described using sodium dodecyl sulfate polyacrylamide gel electrophoresis (SDS-PAGE)^20^,^21^,^26^. Ponceau S staining was used to confirm equal protein loading. Membranes were blocked and incubated with respective antibodies. Thereafter, membranes were incubated with the corresponding secondary peroxidase-coupled antibodies (Santa Cruz-Biotechnology, Santa Cruz, USA).

After enhanced chemiluminescence (ECL; Amersham, Bucks, UK), digital detection was evaluated using Chemi-Smart (PeqLab Biotechnologies, Erlangen, Germany). Data are expressed as means ± standard error of the mean (SEM) with values of controls normalized to 100%.

### Hepatic hydroxyproline content

Analogue segments (200 mg) of snap-frozen murine livers were hydrolysed in HCl (6 N). Hepatic hydroxyproline content was determined photometrically in liver hydrolysates as previously described^26^,^27^.

### Isolation of primary rat HSC and hepatocytes

Primary rat HSCs were isolated as described previously^21^,^26^. In brief, primary HSC were isolated in a two-step pronase-collagenase perfusion from the livers of healthy rats (n = 5) and fractionated by density gradient centrifugation. Viability and purity were controlled and were systematically over 95%. Cells were seeded on uncoated plastic culture dishes. Experiments were performed 1 day after isolation to investigate effects in quiescent HSCs and 7 days after isolation or after the first passage (10 days) when HSCs were fully activated. Primary hepatocytes were isolated from male rats (n = 5) as described previously^28^.

Furthermore, these cells were incubated with the KCa3.1 inhibitor TRAM-34 and activator SKA-31 for 24 hours respectively, and mRNA expression for collagen1, α-SMA and TGFβ were measured. To evaluate effects on cell cycle progression, mRNA expression of cyclin E1 (indicating S-phase initiation) and cyclin A2 (S-phase progression) was measured in 3 different isolations of hepatocytes and HSC incubated with TRAM-34 and SKA-31.

To evaluate effect of KCa3.1 inhibition and activation on cell volume, rat hepatocytes and HSC were incubated with TRAM-34 and SKA-31 for 24 hours. Hepatocyte and HSC cell area representative sections of the cell culture dish were photographed under 20x magnifications using a Nikon Eclipse TS100 inverted microscope with a Nikon DS-Vi1 head camera (Nikon, Düsseldorf, Germany). The measurement of cell area was performed using ImageJ (NIH, USA) and 5–10 cells were analysed per representative section.

### Apoptosis assay

FACS analyses were performed to analyse apoptosis in rat hepatocytes by using the Annexin V Apoptosis Detectin Kit (KD Biosciences, Heidelberg, Germany) as previously described^29^.

### DNA isolation and electrophoresis

DNA from rat hepatocyte samples incubated with agonist, antagonist or left untreated, was isolated using Apoptotic DNA ladder extraction Kit (BioVision, Mountain View, USA) according to the manufacturer’s guidelines. Samples were run in agarose gel, stained with ethidium bromide and visualized by transillumination with UV light, as described previously^30^.

### Histology and immunohistochemical analyses

All analyses were performed on 5 μm paraffin-embedded sections. Positive and negative controls were used to exclude non-specific staining. Immunohistochemistry (IHC) and immunofluorescent (IF) staining of KCa3.1 channels were performed in human samples. In brief, following paraffin removal and dehydration, sections were incubated with a primary antibody (anti-KCNN4 #HPA05384, Sigma-Aldrich, St. Louis, MO, USA). For KCa3.1 IHC we used the PowerVision Poly-Horseradish peroxidase anti-Rabbit IgG (Dako, Glostrup, Denmark) as secondary antibody. For IF protocols we used an Alexa Fluor 488-labeled secondary antibody (Life Technologies, Darmstadt, Germany) for visualization. Finally, sections were counterstained with haematoxylin for IHC or diamidino-2-phenylindole dihydrochloride (DAPI) for IF. Human stained sections were reviewed with an Olympus BX51 microscope and pictures were captured with an Olympus DP26 camera in the cellSens program (Olympus, Hamburg, Germany).

For the detection of collagen fibres in mice, liver samples were stained with 0.1% Sirius red F3B in saturated picric acid (Chroma, Münster, Germany) using standard methods as described previously^27^. Further immunofluorescent staining was performed with collagen1 on murine samples. In human tissue paraffin was removed and sections were dehydrated. Subsequently, sections were incubated with a primary antibody (anti-Collagen, ab34710, Abcam, Cambridge, United Kingdom) and an Alexa Fluor 568-labeled secondary antibody (Life Technologies, Darmstadt, Germany) was used for visualization. Additionally, immunohistochemical analyses were performed for αSMA, which is a surrogate marker for hepatic stellate cell activation. The sections were incubated with a mouse-anti-SMA antibody (Actin clone 1A4; Dako, Hamburg, Germany). Thereafter, a biotinylated goat-anti-mouse (Dako, Hamburg, Germany) secondary antibody was used. Finally, sections were counterstained with haematoxylin.

The histological stainings of the murine samples were digitalised using Panoramic MIDI (3DHistech, Budapest, Hungary). The amount of positive staining was evaluated by computational analysis (Histoquant; 3DHistech, Budapest, Hungary). Large bile ducts and vessels were excluded. The analysis was performed following the principles of computational analysis as described previously^31^,^32^.

### Statistical analysis

Unless otherwise stated, data are given as means ± standard error of mean (SEM) and student’s t-test was used for group comparison when appropriate. Otherwise nonparametric tests were used. Wilcoxon test was used for paired comparison in the same group. Statistical analysis was performed using Prism 5 (GraphPad, La Jolla, USA) software for Mac Os X. P values < 0.05 were considered statistical significant.

## Results

### KCa3.1 channels are upregulated in hepatocytes of fibrotic and cirrhotic human liver

**Figure 1:**
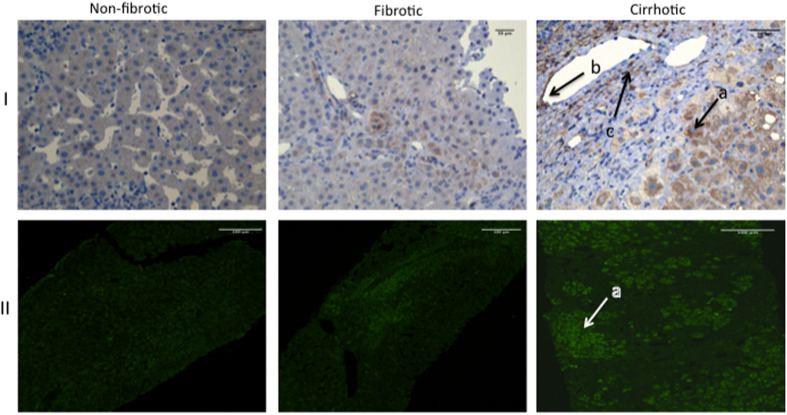
Distribution of KCa3.1 channel in non-fibrotic, fibrotic and cirrhotic human liver. KCa3.1 channel expression evaluated by IHC top panel (I) (x10 magnification) and immunofluorescent stain (II) in bottom panel (x20 magnification). KCa3.1 channels are predominantly expressed in hepatocytes (a) of fibrotic and cirrhotic livers. Furthermore, KCa3.1 was detected in the vessel endothelium (b). To a minor degree KCa3.1 positive staining is seen in the fibrotic septae of cirrhotic livers (c), probably in activated HSCs.

Using immunohistochemical and immunofluorescence stainings, we evaluated the expression of KCa3.1 in non-fibrotic, fibrotic and cirrhotic human liver biopsies (Fig. 1). The corresponding general characteristics of the patients included are summarised in Table 1.

In non-fibrotic livers, regardless of the presence of inflammation, the KCa3.1 channels were only detectable in erythrocytes and vessel endothelium. KCa3.1 was predominantly expressed in hepatocytes of fibrotic livers and were seen to be overexpressed in hepatocytes of cirrhotic livers. In virtually all the hepatocytes KCa3.1 was distributed in the cirrhotic noduli and to a lesser degree in fibrotic septae, (Fig. 1).

### KCa3.1 channels are upregulated in fibrotic livers in rodents

**Figure 2:**
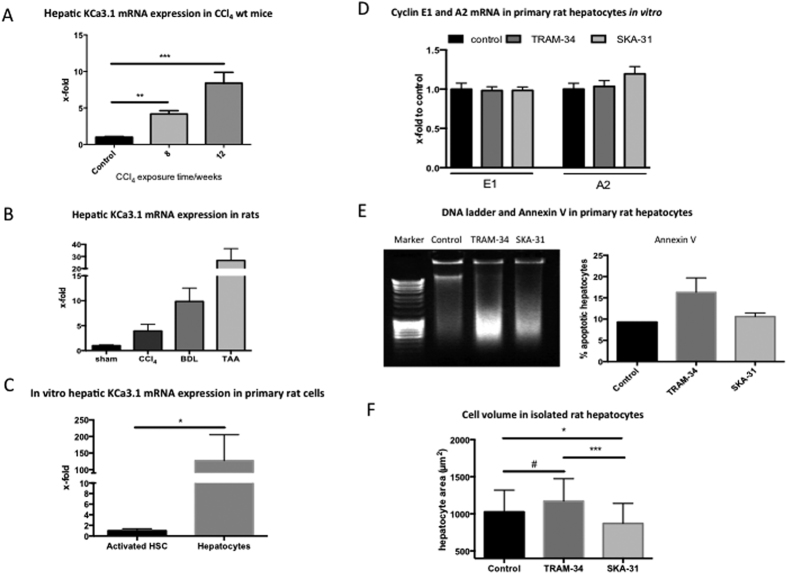
KCa3.1 expression in untreated and fibrotic rodents, cellular differences in distribution and effects. Similar to the differences between normal and fibrotic human livers, KCa3.1 channels are upregulated in fibrotic livers of mice (**A**) and rats (**B**) compared to untreated or sham animals. KCa3.1 is higher expressed in isolated hepatocytes compared to HSC (**C**). Incubation with the KCa3.1 inhibitor TRAM-34 or with the KCa3.1 agonist did not affect significantly the cell cycle progression of hepatocytes (**D**). TRAM-34 directly stimulates hepatocyte apoptosis, as evaluated by increased DNA degradation and annexin V (**E**). KCa3.1 activation with SKA-31 showed no effects on DNA degradation and apoptosis (**E**). KCa3.1 inhibition with TRAM-34 showed a clear trend towards an increase in the hepatocyte cell volume, whereas activation with SKA-31 induced cellular shrinkage (**F**).

To confirm the findings in human tissue samples from diseased livers, KCa3.1 mRNA expression was analysed in mice following 8 and 12 weeks of CCl_4_ induced hepatic injury. Overall, KCa3.1 gene expression increased during liver fibrogenesis and increased with progression of hepatic injury, thereby confirming our data in patients (Fig. 2A). This effect was independent from the fibrosis model, since KCa3.1 expression was also significantly increased in TAA, CCl_4_ intoxicated and BDL rat livers (Fig. 2B).

In addition, *in vitro*, the expression of the KCa3.1 channel was significantly higher in hepatocytes compared to activated HSC (Fig. 2C), thereby validating our findings from immune staining of the human liver.

### KCa3.1 inhibition induces hepatocyte apoptosis and alters hepatocyte volume

To dissect the effects of KCa3.1 in inhibition or activation of the cell cycle in hepatocytes, we measured the gene expression of the cell cycle mediators cyclin E1 (driving the release from quiescence) and cyclin A2 (controlling S-phase progression)^33^. Neither KCa3.1 inhibition with TRAM-34 nor activation with SKA-31 changed cyclin E1 and A2 expression significantly, and hence showed no direct effect on cell cycle progression (Fig. 2D). However, the KCa3.1 inhibitor TRAM-34, but not the activator SKA-31, induced apoptosis of hepatocytes, as shown by DNA ladder formation and FACS analyses of annexin V (Fig. 2E).

Pharmacologic manipulation of the KCa3.1 channel directly affected the cell volume of hepatocytes. Inhibition with TRAM-34 showed a clear trend towards increase in hepatocyte volume, whereas activation with SKA-31 significantly reduced the cell volume (Fig. 2F).

### KCa3.1 channel deficiency aggravates CCl_4_ induced liver fibrosis in mice

**Figure 3:**
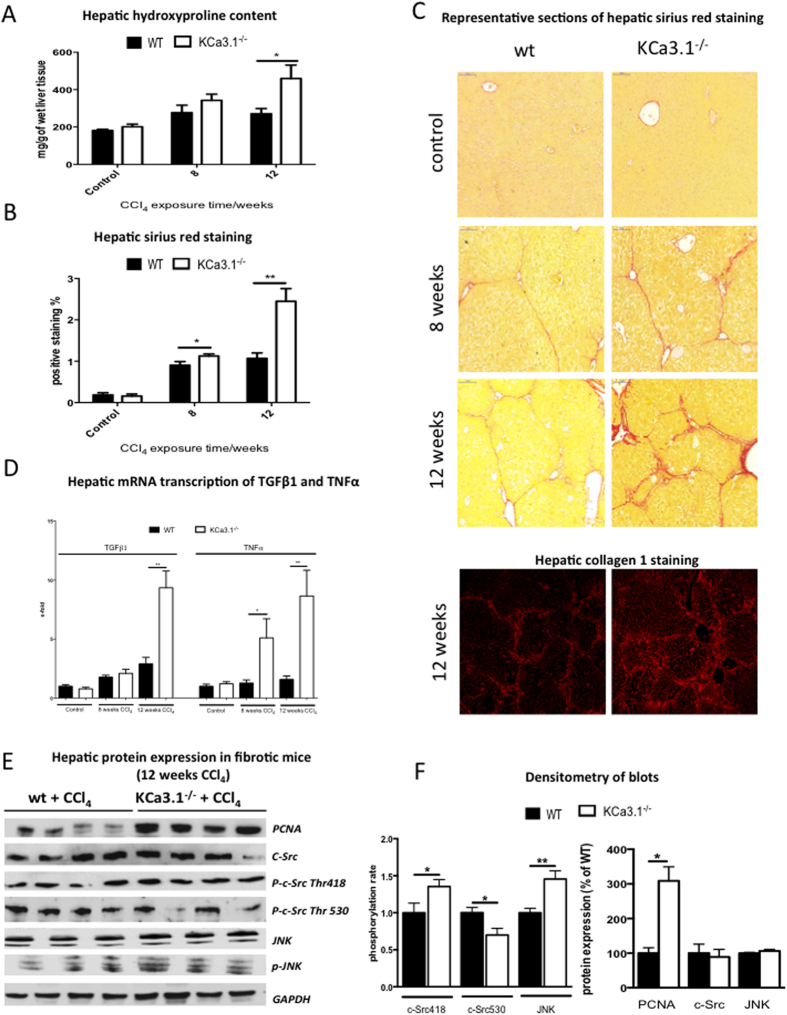
Effect of genetic KCa3.1 depletion in CCl_4_ induced liver fibrosis in mice. KCa3.1^−/−^ mice show increased fibrosis by the evaluation of hepatic hydroxyproline (**A**) and collagen, as evaluated by Sirius red and fluorescent stain for collagen1 (**B,C**) compared to wt littermates. These differences were especially pronounced in severe fibrosis, seen after 12 weeks of CCl_4_ exposure. Similarly, knockouts have higher expression of TGFβ mRNA, and increased inflammatory activity, as evaluated by the higher transcription of the TNFα gene (**D**). Representative Western blot analysis of the hepatic protein-expression of PCNA, JNK, pJNK, p-c-Src Thr418 and 530 were analysed in fibrotic mice following 12 weeks CCl_4_ exposure. The hepatic expression of PCNA was higher in KCa3.1^−/−^. They similarly demonstrated increased pJNK and p-c-SrcThr418 expression, but lower Thr530 (**E+F**), suggesting a direct effect on c-Src and volume control (**E,F**).

The KCa3.1^−/−^ mice showed higher levels of hepatic hydroxyproline (Fig. 3A), and a higher deposition of collagen evaluated by Sirius red staining (Fig. 3B+C) and collagen-1 staining (Fig. 3C). Similarly, the hepatic mRNA levels of TGFβ1 and TNFα were increased in KCa3.1^−/−^ mice after CCl_4_ intoxication compared to their respective wt littermates (Fig. 3D).

Hepatic protein expression of Proliferating Cell Nuclear Antigen (PCNA) was highly increased in KCa3.1 deficient mice compared to wt mice after 12 weeks of CCl_4_ exposure (Fig. 3E+F). Activation, but not expression of hepatic JNK, was significantly increased in fibrotic KCa3.1^−/−^ mice compared to wt littermates (Fig. 3E+F). Moreover, the hepatic protein expression of the c-Src activating phosphorylation (p-c-SrcThr418) was higher, whereas the protein expression of the c-Src deactivating phosphorylation (p-c-SrcThr530) was lower in knockout animals after 12 weeks of CCl_4_ compared to the respective wt mice (Fig. 3E+F). These analyses suggest that proliferation (PCNA) and volume control (c-Src activation) are dysregulated in knockout animals after hepatic injury.

### KCa3.1 inhibition or deficiency stimulates HSC activity *in vitro* and *in vivo*

**Figure 4:**
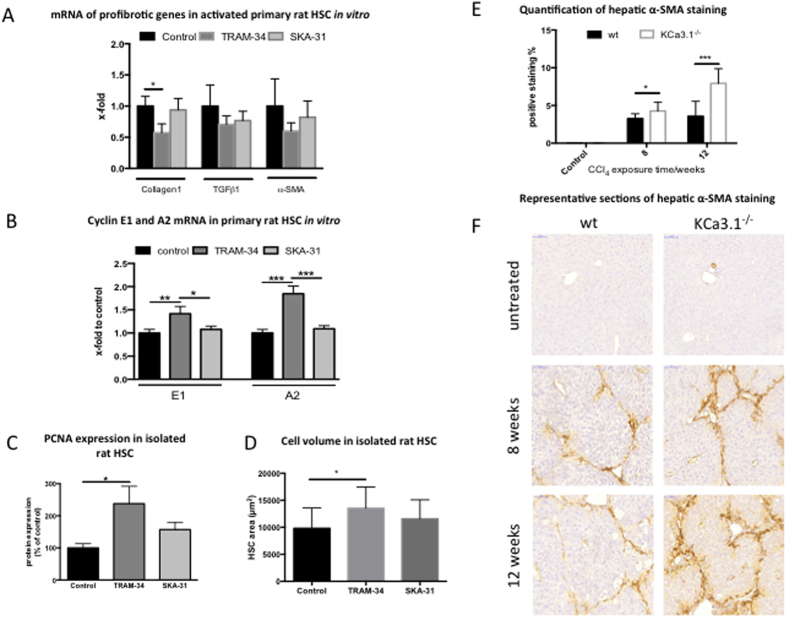
Effect of KCa3.1 deficiency and inhibition on activated HSC. *In vivo*, HSCs were incubated with TRAM-34 and SKA-31 and effect on profibrotic markers evaluated. Incubation with the KCa3.1 inhibitor TRAM-34 suppressed collagen-1 transcription. A similarly trend towards reduced expression was seen for TGF-β. α-SMA was reduced at levels similar to KCa3.1 activation with SKA-31, but was not significantly altered from control (**A**). Incubation with TRAM-34 was followed by an increase in cyclins E1 and A2 in HSCs (**B**), suggesting enhanced cell cycle progression. This was supported by increased protein expression of PCNA in isolated HSC (**C**). Similarly to findings in hepatocytes, TRAM-34 increased HSC volume, but contrary SKA-31 was not able to reduce volume (**D**). CCl_4_ intoxication induces hepatic injury and leads to increased activation of HSCs in Kca3.1^−/−^ mice, as evaluated by α-SMA immunohistochemistry (**E+F**).

To better understand the reason for increased fibrosis in KCa3.1 deficient mice, activated hepatic stellate cells (HSC) were incubated with the KCa3.1 inhibitor TRAM-34 *in vitro*. TRAM-34 significantly suppressed collagen mRNA expression in activated HSC, however no significant effects could be observed on α-SMA and TGFβ mRNA expression (Fig. 4A).

Furthermore, TRAM-34 induced the cyclin E1 and A2 mRNA expression in activated HSC (Fig. 4B), which marks cell cycle progression. KCa3.1 channel stimulation, using SKA-31, however did not affect cell cycle progression (Fig. 4B). PCNA protein expression was increased in isolated HSC after KCa3.1 inhibition with TRAM-34, while activation with SKA-31 did not impact proliferation (Fig. 4C).

Similarly to findings in hepatocytes, TRAM-34 increased cell volume in HSC, but contrary to the hepatocytes, activation with SKA-31 showed no significant reduction in volume (Fig. 4D).

The *in vitro* results were confirmed by the *in vivo* observation of increased α-SMA positive staining, as a sign of increased HSC activation in fibrotic KCa3.1 deficient mice (Fig. 4E+F).

### KCa3.1 inhibition with Senicapoc worsens hepatic fibrosis

Since the genetic deficiency of the KCa3.1 channel might have different effects than the pharmacological inhibition *in vivo*, the KCa3.1 blocker Senicapoc was tested in CCl_4_ induced liver injury in wt mice by oral administration. Senicapoc treated mice ate less than the respective placebo (18,6 g/week vs. 22,9 g/week), but sufficient absorption of the drug was observed in all animals with average p-Senicapoc levels of 587.1 nM.

**Figure 5:**
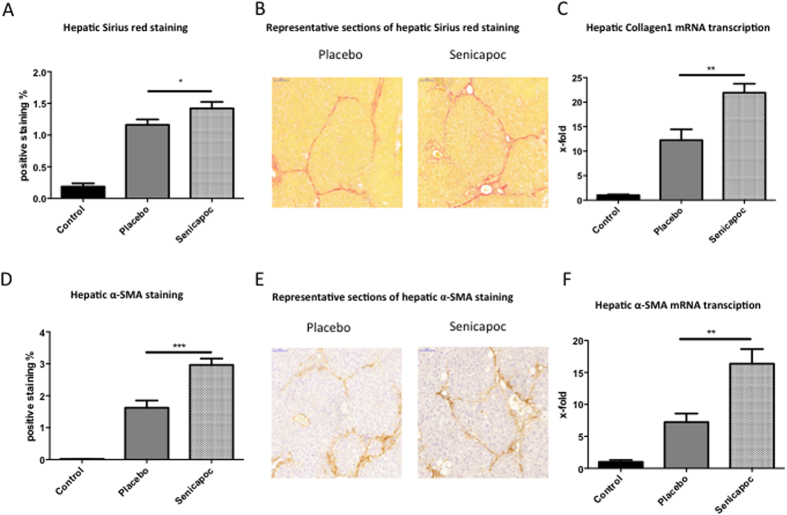
Effects of the KCa3.1 channel inhibitor Senicapoc in CCl_4_ induced hepatic injury in wt mice. Pharmacological KCa3.1 inhibition in CCl_4_ induced liver injury in mice led to increased liver fibrosis compared to wt littermates receiving placebo (**A–C**) and was similarly to the effects seen in KCa3.1^−/−^ mice. Fibrosis was more severe as shown by Sirius red staining (**A+B**) and collagen-1 gene expression (**C**). Senicapoc treatment led to increased HSC activation as shown by immunohistochemiacal staining (**D+E**), as well as by gene expression levels (**F**) of surrogate marker α-SMA.

Senicapoc treated mice showed increased deposition of collagen, as evaluated by Sirius red (Fig. 5A+B), and collagen1 mRNA levels (Fig. 5C). This was associated with higher HSC activation, as assessed by α-SMA staining (Fig. 5D+E) and α-SMA gene transcription (Fig. 5F).

### KCa3.1 inhibition does not alter portal pressure *in vivo*

**Figure 6:**
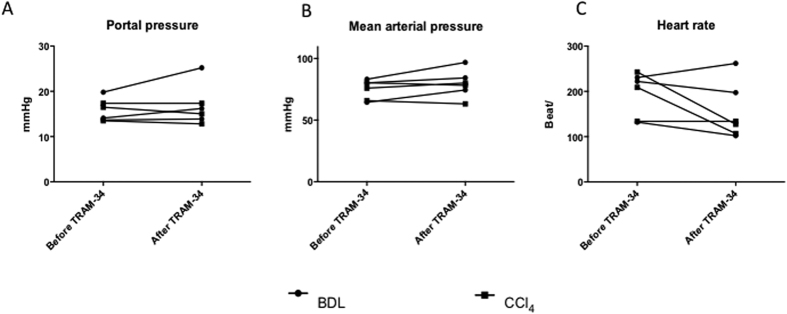
*In vivo* effects on hemodynamics of the KCa3.1 blocker TRAM-34 in bile duct ligated rats. *In vivo*, invasive measurement of portal pressure was performed in BDL and CCl_4_ cirrhotic rats before and after i.v. injection of TRAM-34 (100 nM). Following a 20 minutes observation period, no significant change in portal pressure (mmHg) could be observed (**A**). Measurements of mean arterial pressure (mmHg) and heart rate (beats/min) did not show any alterations compared to prior to TRAM-34 bolus (**B+C**).

Portal pressure measurements were performed to evaluate the direct *in vivo* effect of TRAM-34 on portal hemodynamics in BDL and CCl_4_ cirrhotic rats. However, no significant changes in portal pressure, mean arterial pressure or heart rate were observed (Fig. 6).

## Discussion

This study highlights the importance of the KCa3.1 channel in response to liver injury in humans and rodents. We demonstrate for the first time that the KCa3.1 channel is upregulated in hepatocytes with increasing liver fibrosis, while its absence or inhibition aggravates liver fibrosis.

The KCa3.1 channel is widely distributed in the organism, including the red and white blood cell linage^34^,^35^,^36^, epithelial cells of airway, colon and salivary glands, fibrocytes and vascular endothelial cells^4^,^5^,^6^,^7^,^8^, where as it’s hepatic expression is sparsely explored. A recent histopathological study based on stainings of only a few fibrotic liver samples reported that these channels are upregulated in hepatic fibrosis, and the channels might be located in activated HSC^14^. We confirmed that the KCa3.1 channel is expressed in HSCs. However, KCa3.1 was greatly upregulated in the damaged hepatocytes compared activated HSC of fibrotic livers. Similarly, we showed a significantly higher expression of KCa3.1 mRNA in healthy hepatocytes compared to HSC *in vitro*, however there seems to a discrepancy between mRNA and transcription to protein, as expression of KCa3.1 evaluated by IHC, was absent in healthy human liver. The suggested anti-fibrotic effect of KCa3.1 inhibition in other fibroproliferative disorders has been linked to a suppressed effect on myofibroblast proliferation. *In vitro*, we were similarly able to show suppression of collagen1 mRNA in isolated HSC. However, although the fibrogenic pathways in all fibroproliferative diseases are highly similar, the liver differs from other organs by its unique highly regenerative potential – a capacity linked directly to the physiological roles of the hepatocytes. Upon injury, presence of apoptotic hepatocytes stimulates the inflammatory response and hence fibrogenesis^37^. As KCa3.1 channels in the liver show a high hepatocyte expression compared to the expression seen in HSC, the mediated effect on hepatocytes may blunt any potential anti-fibrotic effect on HSC. The reason why KCa3.1 is found in activated HSC might be the profound changes that occur in these cells showing an increased proliferation, protein expression and migration. KCa3.1 might be one of the requirements of activated HSC to maintain their activated phenotype, since inhibiting it led to proliferation, but not to higher pro-fibrotic activity. The hepatocyte KCa3.1 expression and effect on hepatocyte volume recovery seems important, and explains why the roles of KCa3.1 in the liver differ from effects seen in other parenchymal organs. Therefore, the deficiency or inhibition of KCa3.1 *in vivo* in models of liver injury is associated with hepatocellular damage and inflammation, which drives fibrogenesis, while the mild antifibrotic effects of KCa3.1-inhibition in activated HSC is not relevant in the *in vivo* setting.

The expression of KCa3.1 increased with the severity of liver fibrosis in humans and in three different rodent models of liver fibrosis, suggesting that this is a common pathway irrespective of the species and type of liver injury. Although limited by the small number of animals in the rat experiments, these studies however support the findings from murine and human experiments. The attempt to demonstrate effects of KCa3.1 in a cholestatic mouse model (BDL) was not successful. A pilot study demonstrated a high mortality (>80%) in operated KCa3.1 knockout mice (data not shown), which was linked to a high number of post-operative complications, especially caused by suture rupture induced by the mice – the latter which may be linked to the hyperactive phenotype of KCa3.1 knockouts^17^.

Based on our findings, the exact role of KCa3.1 in hepatic injury and fibrogenesis is unknown. In several cell types (e.g. cancer cell lines, myofibroblasts) channel activation leads to calcium influx, and thereby stimulates cell cycle progression and cellular proliferation^6^,^38^,^39^. In other cell types (e.g. colon epithelial cells, erythrocytes) the channel regulates cell volume. Channel activation induces outflow of chloride and water, which leads to shrinkage^40^,^41^,^42^,^43^. In *in vitro* experiments with hepatoma cells, KCa3.1 regulated the cell volume^3^. This present study clearly supports these findings and shows that KCa3.1 is crucial for the homeostasis of hepatocytes upon injury. Supporting this hypothesis, we have shown that KCa3.1 activation was able to induce shrinkage of hepatocytes, as demonstrated by the reduction in cell volume. Approaches blocking this channel lead to cell cycle dysregulation and apoptosis *in vitro*, whereas the underlying mechanism might be a lack in the capacity to shrink after hepatocyte injury^44^. *In vivo*, activation of JNK and c-Src kinase was increased in KCa3.1 deficient fibrotic mice compared to the wild types assessed by their phosphorylation. It has been shown previously *in vitro* and in the isolated perfused rat liver, as reviewed in^44^,^45^,^46^,^47^, that hepatocyte swelling is directly linked to c-Src kinase, extracellular regulated kinase (Erk)-1/-2 and epidermal growth factor receptor (EGFR) activation in a α_5_β_1_-integrin-dependent manner resulting in hepatocyte proliferation. It is also a well-established concept that hepatocyte cell swelling is linked to proliferation, whereas hepatocyte shrinkage ends up in JNK- and CD95 death receptor-mediated apoptosis^44^,^45^,^46^,^47^. Interestingly, it has recently been reported, that KCa3.1 inhibition in human hepatoma HepG2 cell line cells, which most likely is accompanied by cell swelling, results in apoptosis^48^. Thus it might be an interesting speculation whether an overwhelming hepatocyte swelling in the context of liver regeneration after hepatic injury by either CCl_4_ or BDL in knockouts results in hepatocyte apoptosis and liver fibrosis. This is supported by our findings that in the KCa3.1 *knockout* mice a more pronounced c-Src and an increased hepatocyte injury became detectable. These findings account for a phenomenon associated with swelling since c-Src is usually de-activated in liver fibrosis as shown recently by our group^49^. The abundant KCa3.1 expression seen in human cirrhotic liver tissue may also be explained by a repair mechanism of the hepatocytes, which attempt to restore cell volume in the case of hepatocytes swelling caused by chronic liver injury. Our view is encouraged by recent studies in a model of alveolar epithelial repair in which a complementary role of the swelling activated β_1_-integrin and KCa3.1 was described^50^. This again points to the importance of KCa3.1 in hepatocyte volume homeostasis and differentiated Src family kinase activation^51^ as it might protect hepatocytes from an overwhelming cell swelling in the context of hepatocyte proliferation.

Since the hepatocellular damage is more pronounced in fibrotic mice when KCa3.1 is deficient or blocked, the concomitant increased activation of HSC and fibrosis accumulation is expected. The mechanism by which the HSC get more activated after inhibition of KCa3.1 might be dual. On the one side the hepatocellular damage leads to more hepatic inflammation as shown by TNFα expression; on the other side the blocking the KCa3.1 channel as shown *in vitro* promotes proliferation. Furthermore, it has previously been reported that in quiescent HSC, in contrast to the hepatocytes, addition of death receptor ligands such as CD95L or TNFα are linked to proliferative signalling pathways. This might contribute to the increased activation of HSC after KCa3.1 inhibition as reported in this study, and is probably at least partly JNK-mediated^52^.

HSCs might not be as sensitive as hepatocytes in response to cell volume^47^. Interestingly we found that KCa3.1 inhibition induced swelling in HSC, whereas activation was not able to induce shrinkage in these cells. Therefore, KCa3.1-inactivation might even lead to proliferation of these cells. *In vivo*, these harmful effects of KCa3.1 deficiency or inhibition on liver fibrosis could not be ameliorated by a slight decrease in collagen transcription *in vitro*.

A previous study showed that acute administration of a pharmacological KCa3.1-blocker reduced hepatic resistance in *in situ* liver perfusion experiments^14^. This effect could not be confirmed in our set of *in vivo* experiments. Therefore this is a strict *in vitro* effect without relevance for *in vivo* conditions when evaluating the effects on acute administration of the KCa3.1 blocker. Furthermore, the use of TRAM-34 in experimental settings may be challenged by the lack of selectivity of the blocker towards KCa3.1^53^.

KCa3.1 blockers seem to be beneficial in renal and pulmonary fibrosis or other investigated disorders^6^,^7^,^9^,^11^,^12^,^13^. This study demonstrates, that in liver fibrosis, KCa3.1 expression and proper function is protective for hepatocellular damage and fibrosis. Therefore, the activation of KCa3.1 might be advantageous in the context of liver injury.

Furthermore, the quantification of KCa3.1 expression could be a read-out for hepatocellular homeostasis during liver injury and under anti-fibrotic strategies.

In conclusion, our findings demonstrate that hepatic KCa3.1 activation is an important anti-fibrotic pathway and protects hepatocytes during liver injury and liver fibrosis.

## Additional Information

**How to cite this article**: Møller, L. S. *et al.* The calcium-activated potassium channel KCa3.1 is an important modulator of hepatic injury. *Sci. Rep.* **6**, 28770; doi: 10.1038/srep28770 (2016).

## Figures and Tables

**Table 1:** General characteristics of patients included for immunohistochemical analyses for hepatic KCa3.1 localization and distribution.

Parameters	Non-fibrotic	Fibrosis (F1–F2)	Advanced fibrosis/cirrhosis (F3–F4)
Number	12	20	22
Gender (M/F)	4/8	6/14	15/7
Age, median (range)	42 (20–81)	56 (36–73)	59.5 (45–79)
Child pugh class, n (A/B/C)	n.a.	n.a	14/8/0
Fibrosis stage, median (range)	0 (0–0)	1 (1–2)	4 (3–4)
Inflammation stage, median (range)	0.2 (0–2)	2 (0–3)	1.5 (0–3)
ALT, median (range) U/L	94 (33–325)	135 (19–1210)	33 (6–1310)
Total bilirubin, median (range) μmol/l	6.5 (4–23)	10 (5–539)	20 (7–90)
Alkaline phosphatase, median (range) U/l	122.5 (59–198)	141.5 (70–859)	129 (68–1301)
GGT, median (range) U/l	145.5 (46–801)	266 (54–1495)	194 (43–1401)
LDH, median (range) U/l	224 (113–282)	274 (194–663)	213 (98–420)
Albumin, median (range) g/l	41 (23–46)	41(21–46)	33.5 (22–42)
Creatinin, median (range) μmol/l	65 (53–77)	71 (42–107)	70.5 (50–126)
